# Barriers and Facilitators for Implementation of Palliative Care for Patients with Heart failure – a Rapid Synthesis of Reviews

**DOI:** 10.1007/s11897-025-00715-y

**Published:** 2025-10-24

**Authors:** Thomas Grice-Jackson, Pier Jaarsma, Maria Friedrichsen, Malin Idar Wallin, Everlien De Graaf, Anna Strömberg, Tiny Jaarsma

**Affiliations:** 1 Department of Health, Medicine, and Caring Sciences, Linköping University, Linköping, Sweden; 2 Region Östergötland, Linköping, Sweden; 3 Julius Center Health Sciences and Primary Care, University Medical Center Utrecht, Utrecht University, Utrecht, The Netherlands

**Keywords:** Heart failure, Palliative care, Implementation, Review, CFIR

## Abstract

**Purpose of the Review:**

While the benefits and need for integrating palliative care (PC) services into heart failure (HF) care have been well recognized, integration has been slow. Currently, only a small proportion of patients with HF utilize PC services, meaning there is a discrepancy between the knowledge about optimal care and the implementation of care. In recent years researchers have attempted to understand the barriers and facilitators of implementing PC models to patients with HF which has been captured in both primary, empirical research and secondary literature review papers. We aimed to synthesize these barriers and facilitators to the implementation of PC approaches for patients with HF within a well-known framework for assessing determinants of implementation, the consolidated framework for implementation research (CFIR).

**Recent Findings:**

We conducted a rapid synthesis of reviews using systematic review methods which sought to collate existing review articles pertaining to the research question. We used four search term categories: (1) Heart failure, (2) Palliative care, (3) Implementation, and (4) Review. We conducted searches in four databases (Scopus, EMBASE, CINAHL, COCHRANE) on the 1st of September 2025. We used the CFIR framework to synthesize the data. Firstly, we extracted key barriers and facilitators from the articles. We then categorized this information into sub-constructions of two of the CFIR’s constructs (II. the Outer setting and III. The Inner setting). The search generated 2,080 results, of which 512 were duplicates. After screening, twenty-nine reviews were included in the data extraction and synthesis process.

**Summary:**

A range of barriers and facilitators were highlighted across the reviews. For the inner setting, this included a need for improving communication with patients and between healthcare professionals (HCPs) by engaging more openly and honestly about dying, an increase in joint working as part of multidisciplinary teams, funding and resource issues, and workforce recruitment and training issues. For the outer setting barriers and facilitators were associated with prognostic challenges and the complexity of caring for patients with HF, the needs of HF and geriatric patients, and the evidence and policy landscape associated with the principles of care and implementation of care for HF patients. Despite knowledge about the importance of PC, HCPs will struggle to integrate it into heart failure care unless they address practical, social, cultural, clinical, and economic determinants associated with care. By doing so, health care providers can develop implementation strategies for improving care.

## Introduction

Heart failure (HF) is a common and burdensome condition associated with frequent healthcare utilization, high costs, and up to 50% mortality within five years, despite improvements in therapy over the recent decade [1, 2]. The palliative care (PC) needs of patients with HF are significant, and its clinical benefits are well understood. These benefits include improved quality of life, more stable disease management, and fewer hospital admissions [3]. PC can be delivered by specialist PC professionals in dedicated wards, hospices, or clinics, or through primary care PC models, where PC care is provided by a range of primary care professionals with PC training in community or home-based settings. While most PC is delivered in specialist settings such as hospices or palliative wards, primary care PC is increasingly being adopted to complement specialist care, reduce strain on specialist PC services, and provide earlier access to PC in a patient’s disease trajectory, with the additional advantage that primary care by nature already embraces a generalist multidimensional approach [4].

Another emerging PC model in HF care is embedded PC, in which PC professionals are integrated into other healthcare teams to provide care in settings where patients are already receiving treatment [5, 6].

Despite the known benefits, the uptake of PC services for HF patients remains low compared to other life-limiting illnesses with a similar life-limiting prognosis, such as cancer [7]. Additionally, HF patients who do use PC often do so at a late stage of their disease trajectory [8]. This discrepancy suggests that implementation determinants may be influencing the use of PC. Implementation refers to the contextual and organizational factors that exist outside an intervention or program’s design but significantly impact its success [9]. Several implementation frameworks have been developed to support researchers in assessing and evaluating implementation. Among them, determinant frameworks specify barriers and facilitators that influence outcomes [10]. One such framework is the Consolidated Framework for Implementation Research (CFIR) [11], which consists of five domains: (I) the innovation domain, (II) the outer setting, (III) the inner setting, (IV) the individual domain, and (V) the implementation process domain. Each of these domains contains multiple subconstructs. While CFIR is conventionally used to assess interventions, it can also be applied to appraise a given field of research [12].

Barriers and facilitators for PC delivery were recently summarized in an umbrella review [13]. While this review included some studies focused on HF patients, the majority examined other conditions—mainly cancer—or did not differentiate between conditions. PC for HF patients presents unique challenges and considerations [3]. Therefore, implementation strategies aimed at improving care must be tailored to the specific needs of patients, informal caregivers, and relevant stakeholders. Although several existing reviews discuss barriers and facilitators to PC delivery across various care settings including HF, to our knowledge, none have used implementation science methods, tools, and frameworks to synthesize and evaluate findings. Implementation science frameworks are crucial for conducting robust, generalizable, and impactful research as they provide the necessary structure to guide the research process, facilitate communication, and ensure that invention determinants are properly identified, implemented, and evaluated [14]. Furthermore, the review papers that examine the impact of different PC models (specialist, primary, or embedded) on the delivery of PC for HF patients highlight only a small number of studies and provided limited evidence regarding their impact on patient outcomes [15].

This review aims to explore the implementation determinants associated with PC for HF patients using the CFIR framework. Qualitative syntheses of reviews compiles and analyses findings from multiple review articles within a subject area, allowing for a comprehensive understanding of common themes and patterns [16]. This work has been carried out to support the early stages an implementation evaluation as part of a European research collaboration named RAPHAEL (Integ**RA**ting a **P**alliative Care Approac**H** for P**A**tients with H**E**art Fai**L**ure - Home - Raphael). It represented as the first stage in identifying potential implementation barriers and facilitators which would be further developed and refined before being used to identify implementation strategies. The project, including this review, is using the Consolidated Framework for Implementation Research (CFIR) to structure much of the implementation analysis [11]. We therefore adopted a ‘rapid review’ approach to provide a timely and concise snapshot of existing research [17, 18]. As a result, the goal of this research is to systematically identify existing reviews which represent the current state of knowledge regarding implementation and integration of PC models for HF patients.

## Methods

### Research Question

What are the barriers and facilitators to the implementation of PC approaches and models for patients with HF?

### Design

The design for this review was informed by rapid review methods in which components of the systematic review process are simplified or omitted to produce information in a timely manner [17, 18]. A summary of the methodological simplifications/omissions from the systematic approach are summarized in appendix 1. The articles included in the rapid review were synthesized using the Consolidated Framework for Implementation Research (CFIR) as a deductive coding framework to identify barriers and facilitators [11].

### Search strategy

 We conducted a systematic search for relevant literature on the 1 st of September 2025 across four databases: Scopus, EMBASE, CINAHL, and Cochrane (title, abstract, keyword searches). We developed the search terms based on several related preexisting reviews [19–21] (see appendix 2). We restricted the search to articles published in English.

### Study Management and Inclusion Criteria

We imported the search results into Rayyan for screening. After duplicate removal, one researcher (TGJ) assessed the titles and abstracts and full texts of all studies against the inclusion and exclusion criteria (see appendix 3). At both screening stages the results of screening were calibrated by two researchers (PJ, TGJ) by assessing included articles and a selection of excluded articles against the study’s inclusion/exclusion criteria. During abstract and Title screening and full text screening several articles were initially listed as “maybe” by the lead author. These articles were a particular focus of calibration, and a meeting was held to discuss disagreement. The final included texts after full text screening were then reviewed once again by the two authors involved in screening (TGJ, PJ) and disagreements on inclusion were resolved by a separate author (TJ).

### Data Extraction

Extracted data included the author and publication year, type of review, country of origin, number of included studies, patient population, PC models and approaches (specialist, primary, integrated, or embedded), study aims, key findings, facilitators to implementation, and barriers to implementation. We did not perform a quality appraisal or risk of bias assessment.

### Data Analysis

We used a framework analysis approach to analyze the literature [22]. Following data extraction, we categorized and synthesized key facilitators and barriers to implementation using the Consolidated Framework for Implementation Research (CFIR) [11].

The CFIR framework is primarily designed to evaluate specific interventions or programs, and several of its constructs are less suited to reviewing broader literature. For example, the Innovation construct of CFIR refers to the specific design of a given intervention, the Individuals construct pertains to the staff involved in delivering an intervention or program, and the Implementation construct relates to the specific activities and strategies used to implement an intervention or program. For this analysis, we used only the Inner Setting and Outer Setting constructs, as they address social, economic, cultural, organizational, and legal implementation determinants that are not tied to a specific intervention or program. The Outer Setting refers to contextual factors outside healthcare delivery organizations that influence the implementation of care. The Inner Setting pertains to the environment in which PC is provided, encompassing implementation determinants within a healthcare organization’s control, remit, and responsibilities. We categorized the data into the five subconstructs of the Outer Setting and the nine subconstructs of the Inner Setting. We found no substantial relevant data for the remaining constructs.

## Results

### Study Characteristics

**Fig. 1:**
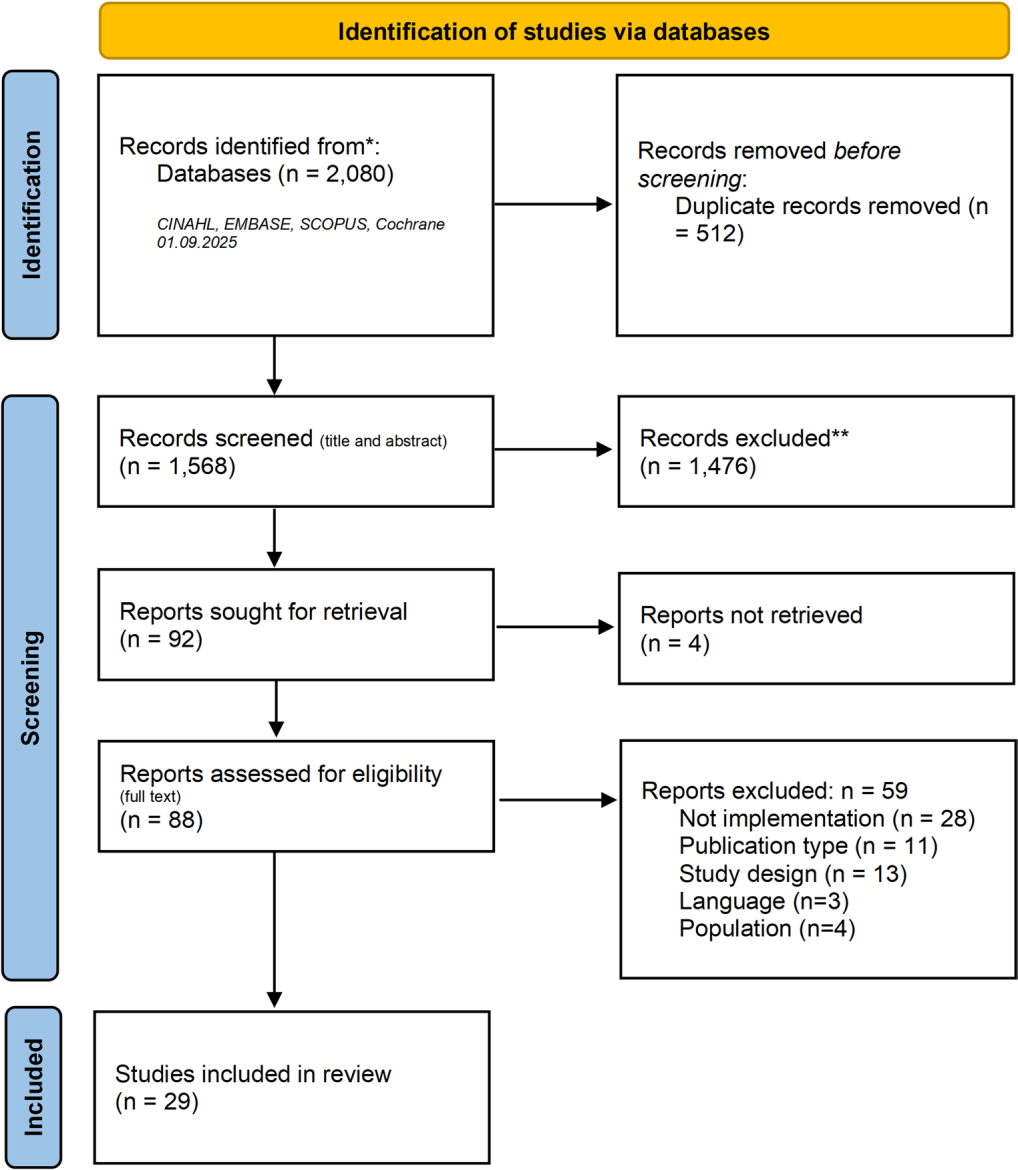
PRISMA flowchart of the search and screening process

Our search generated 2,080 articles, of which 512 were duplicates. After initial title and abstract screening, 1,479 articles were removed, and four full texts could not be retrieved. Eighty-eight articles were included for full-text screening, of which fifty-nine were excluded for not meeting the inclusion criteria (appendix 3). A total of twenty-nine reviews were eligible for inclusion and were incorporated into the synthesis (see Fig. 1 for the PRISMA flow diagram; [23]. Table 1 provides a summary of the characteristics of the reviews included. The reviews were published between 2007 and 2025, and during our analysis, we will provide commentary on the maturation of the field where relevant.

**Table 1:** Characteristics of included articles

Author	Year	Journal	Methodology	No. articles	Palliative care models	Countries represented
Stuart [24]	2007	Current Opinion in Supportive and Palliative Care	Non-systematic integrative review	Not stated	Multidisciplinary	Not stated
Lemond & Allen [25]	2011	Progress in Cardiovascular Diseases	Position paper and literature review	Not stated	Specialist	Not stated
Low et al. [26]	2011	Journal of Cardiac Failure	Systematic integrative review	48	Primary and specialist and multidisciplinary	USA, UK, Sweden, The Netherlands, Spain, Canada, Ireland, Australia, and New Zealand
Momen & Barclay [27]	2011	Current Opinion in Supportive and Palliative Care	Systematic integrative review	23	Specialist	Not stated
Dalgaard, Bergenholtz, Nielsen, & Timm [28]	2014	Palliative and Supportive Care	Systematic scoping review	44	Specialist	Germany, Australia, Netherlands, USA, Canada, UK,
Fernando, Percy, Davidson [29]	2014	Current opinion in Supportive and Palliative Care	Non-systematic literature review	Not stated	Primary PC, Community-based, Telehealth	Not stated
Gelfman, Kalman & Goldstein [30]	2014	Journal of Palliative Medicine	Non-systematic literature review	Not stated	Primary and specialist	Not stated
Clement, Painter & Shaffer [31]	2016	Current Cardiovascular Risk Reports	Non-systematic integrative review	Not stated	Specialist and community based	Not stated
McIlvennan & Allen [32]	2016	The British Medical Journal – State of the Art Review	Non-systematic integrative review	Not stated	Primary, specialist, tertiary	Not stated
Gelfman et al. [33]	2017	Heart Failure Review	Non-systematic literature review	Not stated	Primary and specialist	Not stated
Asano et al. [34]	2019	Collegian	Systematic integrative review	43	Multidisciplinary	UK, USA, China, Netherlands, Sweden, Canada, Australia, Spain, Germany, South Korea, Japan, Kenya
Schichtel, Wee, MacArtney & Collins [35]	2019	British Medical Journal	Systematic review	17	Advance care planning	UK, Australia, Canada, Sweden, USA, Belgium
Wiskar, Toma, Rush [36]	2018	Trends in Cardiovascular Medicine	Non-systematic literature review	Not stated	Primary, specialist and multidisciplinary	Not stated
Singh et al. [37]	2019	Heart, Lung, and Circulation	Integrative literature review	26	Primary, specialist and multidisciplinary	US, UK, Belgium, Germany
Thompson & Moser [38]	2020	Heart & Lung	Systematic review and qualitative synthesis	6	Specialist	Not stated
Williams, Kozachiik, Karlekar & Wright [39]	2020	American Journal of Hospice & Palliative Medicine	Systematic integrative review	6	Advance care planning	Not stated
Bayly et al. [40]	2021	British medical journal - Open	Tertiary Systematic Review	78	Primary, specialist, and community	USA, Europe, Southeast Asia, Oceania, Africa
Crimmins, Elliott, & Absher [41]	2021	American Journal of Hospice & Palliative Medicine	Systematic integrative review	12	Primary palliative	Not stated
Godfrey, Kirkpatrick, Kramer & Sulisto [42]	2023	Frontiers in Cardiovascular Palliative Care	Position paper and literature review	Not stated	Multidisciplinary	Not stated
McConnell et al. [43]	2023	Open Heart	Systematic realist synthesis	130	Multidisciplinary	USA, UK, Europe
Nunciaroni et al. [44]	2023	American Journal of Hospice & Palliative Medicine	Systematic realist synthesis	14	Multidisciplinary	Brazil. USA, South Korea, Sweden, Netherlands, Ireland, Japan
McClung, Frishman & Aronow [45]	2024	Cardiology in Review	Non-systematic literature review	Not stated	Multidisciplinary	Not stated
Chuzi and Manning [46]	2024	Current Opinion in Cardiology	Non-systematic narrative review	7	Primary, secondary, multidisciplinary	USA and Hong Kong
Murray et al. [47]	2024	ESC Heart Failure	Systematic review	12	Specialist	USA, Canada, Sweden, Netherlands, Australia, South Korea, UK
Agho et al. [48]	2025	Cureus	Systematic literature review	18	Specialist, Primary, community	International (unspecified)
Allcroft et al. [49]	2025	ESC Heart Failure	Narrative review with realist analysis CMO analysis	21	Primary, secondary, multidisciplinary, community	USA, UK, Hong Kong, Sweden, China, Singapore
Caleffi et al. [50]	2025	Healthcare	Systematic review using Theoretical Domains Framework (TDF)	26	Primary, secondary, multidisciplinary, community, hospice	UK, Australia, Germany, Iran, USA, Hong Kong, Ireland, South Korea
Perera et al. [51]	2025	Palliative Medicine	Scoping Review	13	Primary, secondary, multidisciplinary, community	Europe, North America, Asia
Peng, Lu, and Mo*l.* [52]	2025	British medical journal - Open	A systematic review using TDF and COM-B	48	Community-based	International (unspecified)

Regarding the geographic distribution of articles included in the reviews, most reviews included articles which were conducted in Western countries with the USA being the most widely represented country in the reviews. European countries were also regularly represented in the reviews, particularly UK, Germany, the Netherlands and Sweden. Asia countries were somewhat represented in the reviews although particularly though with a Western leaning academic landscape (Japan and South Korea). It is notable for example that very few reviews included studies carried out in China. Australia was also relatively widely represented in the reviews and very few studies were included from south America and Africa. Fourteen of the review articles did not state their geographical representation.

## The OUTER Setting

We extracted data for five subconstructs in the Outer Setting and presented descriptions of the barriers and facilitators for each subconstruct in Table 2. The text below includes numerical citations to the articles referenced in the analysis.

**Table 2:** OUTER settingouter setting: the setting in which the inner setting exists, e.g., hospital system, school district, state. there May be multiple outer settings and/or multiple levels within the outer setting (e.g., community, system, state)

SUB-CONSTRUCT	FACILITATORS	BARRIERS
Local Attitudes Sociocultural values (e.g., shared responsibility in helping recipients) and beliefs (e.g., convictions about the worthiness of recipients) encourage the Outer Setting to support implementation and/or delivery of the innovation.	• Complimentary faith-based and spiritual support [31, 40, 48, 51]	• Patients commonly underestimate disease severity and overestimate prognosis [36, 45, 50] • Patient misperceptions of PC as a ‘near death service’ or ‘giving up’ [37, 41, 43, 50] • Patients do not want to discuss death [29, 34, 40, 41, 44, 50, 52] • Patient spiritual and cultural attitudes and beliefs [44] • ICD deactivation considered taboo by patients [47]
Local Conditions Economic, environmental, political, and/or technological conditions enable the Outer Setting to support implementation and/or delivery of the innovation.	• Increasing evidence, example of good practice, and research agenda [30, 40, 43]	• Lack of research consensus related to models of care [33, 36, 48] • Lack of clear evidence base about implementation [32, 33, 40, 48] • A lack of established guidelines for ICD management [47, 50]
Patient needs and resources The extent to which patient needs, as well as barriers and facilitators to meet those needs, are accurately known and prioritized by the organization.	• PC supports patients emotional and psychological needs [48, 52] • Educating Patients about benefits of PC [47] • Including family members in decision making [44]	• Complex disease trajectory [25, 28, 31, 32, 34, 35, 41–45, 48, 50–52] • High levels of demand [38] • HF symptoms complicate care [25, 34] • Challenges with geriatric needs [31] • Emotional needs [26, 27, 35, 47] • Implanted devices [26, 32, 36, 43, 47] • Social deprivation, urban rural divide, and ethnic/cultural background impacts care [29, 31, 40, 44, 45, 47]
Policies and Laws Legislation, regulations, professional group guidelines and recommendations, or accreditation standards support implementation and/or delivery of the innovation.	• Clear national guidelines for monitoring ongoing patient needs [28, 42] • Clear no resuscitation laws [50] • Guidelines for ICD deactivation [47]	• Lack of policies and clinical governance [40, 50] • Knowledge of the legal issues [35, 41] • Ethical issues associated with deactivation of cardiac device [38, 41, 47]
Financing Funding from external entities (e.g., grants, reimbursement) is available to implement and/or deliver the innovation.	• PC associated with lower healthcare costs [48]	• Financial and administrative models [32, 34, 36] • Limited health care budgets [40, 41, 49, 50]

### Individual Attitudes

The reviews primarily focused on patient, family, and caregiver values, beliefs, and perceptions as barriers to implementation. Patients’ expectations about their prognosis sometimes lead them to feel that the introduction of PC is inappropriate or premature [24–26]. The reviews also highlighted misperceptions of PC services as being solely for ‘end of life’ (EoL) care or as an indication that providers were “giving up” on them [26–29]. Spiritual, religious, and cultural values were identified as both barriers and facilitators to PC provision. For some patients, these values contributed to an aversion to discussing PC when nearing death [25, 26, 29, 30]. However, complementary faith-based or spiritual support was also found to enhance the effectiveness of PC [30–33]. Over the 19-year span of these articles, there has been little change in the reporting on patient perceptions and attitudes toward PC.

### Local Conditions

Three of the articles recognized that increasing evidence regarding the efficacy of PC and examples of good practice stimulated integration of PC services and meant that the issue was raised further up the research agenda [28, 32, 34]. However, the lack of research consensus about specific models of care [24, 33, 35], as well as a lack of evidence regarding the implementation of PC services, acted as a barrier to implementation [27, 33, 34, 36]. There are some signs that this lack of evidence is changing in the field. The earlier reviews that discuss the evidence base, carried out between 2014 and 2018 [24, 34–36], highlight a lack of evidence. However, the most recent reviews note that there is a growing evidence base of good practice and that the field is becoming a larger priority amongst healthcare researchers [26, 28, 30, 32, 33, 37, 38].

### Patient Needs and Resources

The most common challenge was the complexity of the HF trajectory, which makes PC planning and prognostication challenging for care providers [24, 26–31, 33, 36, 38–46]. This complexity is further increased for patients with cardiac devices, such as a defibrillator, who may face an increased likelihood of a prolonged death due to an increased risk of pump failure or the risk of hospital admission instead of a sudden cardiac death [24, 28, 36, 47, 48]. The views also highlighted the benefits of PC in terms of providing patients with emotional and psychological support during their care [30, 37]. Furthermore, the importance of educating patients in the benefits of PC was also highlighted as a facilitator [48].

One review also highlighted the high levels of demand for PC in countries where PC is a medical specialty and where it is not integrated but provided by specialized HCPs. A lack of resources can lead to a barrier in providing PC, particularly when patients use PC services over an extended period [45]. Additionally, several common symptoms of HF, such as fatigue, cognitive decline, and functional decline, can act as barriers to the communication, planning, and monitoring required for effective PC [40, 43]. One review also pointed out that challenges associated with general geriatric patients, such as comorbidity or functional and cognitive decline, are also relevant to patients with HF because the majority are older adults [31].

### Policies and Laws

Where they existed, national guidelines on symptom monitoring for patients with HF were discussed in the reviews as a facilitator of PC [42, 46]. However, one review discussed the current lack of existing guidelines [32, 38]. The ethical and legal complexity associated with advanced care planning, living wills/advanced decisions, and no resuscitation orders to refuse treatment was discussed as a barrier to PC, as these can make HCPs cautious or averse to making decisions related to PC for patients with cardiac devices [28, 38, 44, 45].

### Financing

One review highlighted the cost-saving potential to effective home-based PC compared to convention care due to lower rehospitalizations [37]. However, several review papers discussed the difficulties of implementing PC due to limited healthcare budgets at regional or national levels, which limits the necessary expansion or development of services [27, 32, 38]. For primary PC, the economic benefit remains unclear, as most studies to date have focused on inpatient PC costs [24]. There are also challenges associated with who should pay for PC services in financial and administrative national or regional systems that rely on out-of-pocket expenses or private insurance [25, 36, 43].

## The INNER Setting

We extracted data for nine subconstructs in the Inner setting, and presented barriers and facilitators extracted from sub-constructs in Table 3. The text below includes numerical citations to the articles referenced during the analysis.

**Table 3:** INNER settingthe setting in which the innovation is implemented, e.g., hospital, clinical, community setting. There May be multiple inner settings and/or multiple levels within the inner setting, e.g., unit, classroom, team

SUB-CONSTRUCT	FACILITATORS	BARRIERS
Structural Characteristics Infrastructure components support functional performance of the Inner Setting. Note: Use this construct to capture themes related to Structural Characteristics that are not included in the subconstructs below.	• Action plans and referral plans [29, 34, 47, 49, 51] • Integrated PC and cardiology programs [28–30, 51] • Established clinical responsibility [26] • Early intervention and timing [47–49, 51]	• Patient preferences do not align with delivery models [34] • Lack of formalized deactivation plans for implanted devices [26, 32, 46, 48] • Poor geographic accessibility. [31] • Unclear referral criteria [31, 34, 42] • Siloed/fragmented care [32, 45, 51]
Relational Characteristics There are high quality formal and informal relationships, networks, and teams within and across Inner Setting boundaries (e.g., structural, professional).	• Multidisciplinary teams and primary PC [24, 26, 28, 34, 37, 42, 46, 49–52] • Collaboration on shared initiatives [26, 28] • Psychosocial support for HCP [40, 50, 51] • Effective communication between HCP [35, 38, 44, 50] • Sharing information teams [24, 32, 35] • PC strengthened by nurse leadership [50] • Interdisciplinary mentoring improve collaboration and training [51]	• Late referrals from cardiology teams into PC, [28, 42] • Poor inter-professional communication and collaboration [26, 35, 45, 48] • Lack of clarity about roles and who is responsible [26, 30, 35, 43, 44, 50, 51]
Communications There are high quality formal and informal information sharing practices within and across Inner Setting boundaries (e.g., structural, professional).	• Frequent, close, and respectful two-way communication [26, 27, 29, 34, 35, 38, 44, 50] • Healthcare professional initiation of a conversation about death and dying [34, 35] • Establishing the goals of care for patients [31, 35, 50] • Early communication and intervention about PC options [29] • Identifying a patient’s preferences and values [35, 51] • Emotional support for patients [25, 36, 44] • Communication of PC values [36] • Shared decision-making [32, 35, 39, 40, 44]	• Lack of communication with patients [24, 28, 30, 31, 34, 37, 41] • HCP’ difficulty in approaching discussions relating to death [24, 26, 27, 34, 40, 41] • HCP have limited understanding of patient preferences [26] • Patients often find it hard to get information [38]
Culture There are shared values, beliefs, and norms across the Inner Setting. Note: Use this construct to capture themes related to Culture that are not included in the subconstructs below.	• Taking a “hope for the best, plan for the worst” culture [30] • Family physicians or primary care services [26]	• PC is perceived to be a ‘near death’ [24, 27, 30, 32, 37, 40–42, 50] • Death denying attitudes amongst HCP [30, 40] • A culture of avoiding PC [30, 41, 46, 47, 50] • A fear of losing hope, accepting death or failing amongst patients [27, 35, 36] • Differing care philosophies between PC and cardiology [26, 43, 50, 52] • PC nurse led whilst Cardiology physician led [52]
Tensions for Change The current situation is intolerable and needs to change.	• Establishment of PC champions [30] • Incentives for and performance measures [32, 50, 52]	• Current care is reactive [41]
Relative priority Implementing and delivering the innovation is important compared to other initiatives.	• Non-identified	• Healthcare professional focus on curative intervention [24, 34, 43, 45] • Competing demands in healthcare professional’s clinical work. [35, 47]
Mission Alignment Implementing and delivering the innovation is in line with the overarching commitment, purpose, or goals in the Inner Setting.	• Patient centred care [25, 27, 31, 39, 40, 52] • Goal setting [25, 40] • Community-based and Home-based care. [40, 50, 51] • Shared decision making [35, 39, 47]	• Non-identified
Available resources Resources are available to implement and deliver the innovation. Note: Use this construct to capture themes related to Available Resources that are not included in the subconstructs below.	• Adequate staffing and finance support [30] • Protected time for PC consultations [35, 43] • Providing a range of treatment options [35] • Reminders to consider end-of-life conversations [35]	• Lack of trained PC professionals and workforce gaps [29, 31, 33, 35, 36, 41, 45, 48, 50, 52] • Lack of time to see patients [26, 27, 41, 42, 50, 52] • Lack of screening procedures and referral guidelines [26, 28, 30, 34–36, 41, 42] • Lack of space for PC appointments [48, 50] • Inconsistent use of telehealth technology [48, 50] • Underutilized PC teams [46, 51]
Access to Knowledge & Information Guidance and/or training is accessible to implement and deliver the innovation.	• Ongoing education of cardiology HCP about PC [33–35, 40, 43, 44, 48, 50, 52] • Education for PC professionals on HF needs [33, 37] • Training on emotional and intellectual demands of PC [32] • Multi- professional training [46, 47, 49]	• Lack of awareness about PC treatment options, amongst cardiologists [26, 32, 35–38, 41, 45, 50–52] • Lack of trained PC workforce [33, 37, 42] • Inadequate communications training [25, 35, 42] • Lack of knowledge about HF amongst PC professionals [26, 47] • Lack of legal and ethical knowledge [47]

### Structural Characteristics

Several reviews highlighted the value of working in multidisciplinary teams of HF and PC clinicians who work together, share case management, and use integrated programs/initiatives in which different models are provided in parallel [26, 27, 39, 42, 47, 49]. Two reviews highlighted that siloed or fragmented PC and cardiology care is often a barrier to providing good care [36, 47, 49].

Structural characteristics also included processes and systems used by care organizations to facilitate care. For example, the use of PC action plans, in which patients plan goals and wishes for their care with an HCP, is highlighted as a facilitator of care [43, 49]. Additionally, clear referral and assessment criteria, as well as integrated scheduling systems between PC and cardiology, can act to improve care [26, 43, 49]. Several reviews highlighted that the lack of these processes, especially for patients with a cardiac device, is a barrier to care [26, 31, 36, 43, 46, 48, 50]. Over time, the discussion of structural characteristics moved from broader discussions of the advantages of joint working and collaboration towards more detailed discussions of the issues faced when engaging in joint working, such as scheduling systems, referral criteria, and use of eHealth and telehealth. This may indicate that the field is focusing more on the practical issues of implementation.

### Relational Characteristics

Frequent and close joint working in multidisciplinary teams made up of professionals in cardiology and PC professionals was highlighted as a facilitator of good care [26, 29, 30, 33, 38, 39, 42, 43, 45–47], which could be improved through the implementation of shared initiatives [42, 47]. These reviews also highlighted the value of primary PC services, which make use of multidisciplinary care teams to provide PC care to patients earlier in their illnesses [34, 35].

Sharing information between primary and secondary healthcare teams to identify appropriate early intervention for PC advanced care planning was considered a facilitator of care [36, 39, 44], as was the establishment of clinical responsibilities for communicating with patients about PC referrals [26, 28, 29, 34, 38, 44, 47]. Additionally, mentoring of colleagues between palliative care and cardiology teams acts as a facilitator in improving collaboration and training across disciplines [26]. Discussions regarding relational characteristics between HCPs have remained stable over time, between the early and more recent reviews in this analysis.

### Communication

Frequent, close, and respectful two-way communication with family members and patients was considered a facilitator of good care [26, 29, 30, 41, 43, 44, 47, 49, 50], while a lack of communication with patients can lead to poor expectations about what PC involves and later referrals to PC [27, 32, 35, 39, 43]. The reviews considered it important for HCPs to initiate early and open conversations about death and dying and to establish clear goals of care through the identification of patient preferences and needs [29, 31, 34, 35, 51]. Providing emotional support to patients and communicating the values and principles of PC was also seen as a facilitator [24, 29, 42]. Involving the patient and informal caregivers to identify appropriate support through shared decision-making was also highlighted as a way of improving patient experiences of care [29, 32, 36, 44, 50].

HCPs’ difficulty in approaching discussions relating to death was considered a barrier, as this kind of communication can be seen as challenging or taboo by some outside of PC [27, 32, 39, 41, 43, 50]. One review highlighted that it is often difficult for HF patients to get information about PC from HCPs [45], and another review highlighted that some physicians wait for the patient to initiate EoL conversations [44]. Some patients also wanted to avoid conversations about death, meaning that approaching the topic is often difficult for HCPs [28, 29, 32, 43, 49]. One review noted that primary care services could be well suited to initiating EoL discussions because they often have pre-existing relationships with patients [47].

Several of the reviews highlighted poor interprofessional communication and collaboration as a barrier to care [24, 44, 47], which often meant that patients were referred too late from cardiology services into PC [42, 44, 47]. According to one review, there is a lack of clarity about who is responsible for initiating palliative conversations [28, 29, 35, 44, 47]. Discussions regarding patient communication have remained stable over time, between the early and more recent reviews in this analysis.

### Culture

Amongst many clinical professions, including cardiology, PC is perceived to be a ‘near death’ or ‘cancer only’ service [27, 29, 34, 36, 38, 39, 46, 51]. Different care philosophies between key specialties treating advanced HF patients (cardiology and specialist PC) were seen as a barrier to good PC delivery [28, 47]. The culture of PC being seen as a near-death service has remained consistent over the time in which these reviews were published. Although the difference in care philosophies between specialties applied to all professional staff groups, this was most evident among physicians. Cardiologists felt that they were poor at recognizing PC needs in their patients and felt a sense of failure when patients deteriorated [47].

The reviews highlighted a belief amongst HCPs that patients would fear losing hope or accepting death, and that PC care would be considered a failure of care for patients [24, 41, 44, 46]. This culture can mean that PC is only provided late in the HF trajectory [28, 42]. Additionally, one review highlighted a perception that other colleagues are more appropriate for EoL conversations [44]. Several reviews also highlighted a tension between the care philosophies of cardiology, which is about intervening and preserving life, and palliative, which concerns managing symptoms and accepting death [28, 30, 38, 47].

As for facilitators, the reviews highlighted that the culture of “hope for the best, plan for the worst” can facilitate PC care [34]. This review also highlighted that integrating PC into the model of HF care means a rethinking for both PC and HF practitioners alike, and that this type of culture change can be a slow process.

### Tensions for Change

One review pointed out that current PC for patients with HF is too reactive and is not meeting the needs of patients and staff, arguing that this indicates a need for change [27]. Creating incentives for skills training and exercise of PC principles among cardiologists, such as offering continuing medical education credits regardless of specialty, can help bridge the gap in knowledge within cardiology teams [30, 36, 38]. Another facilitator to establishing a collaborative relationship is identifying a PC champion within the cardiology teams [31]. A PC champion is a healthcare professional who advocates for and supports best practices in PC within their workplace, helping colleagues integrate compassionate, patient-centered approaches to improve quality of life for people with serious illness. This role could be official or non-official (i.e. a HCP with a passion for advocating for PC) and can help to ‘translate’ the PC paradigm to HF clinicians and vice versa.

### Relative Priority

Several review papers highlighted the differing priorities between professionals, with cardiologists focusing on curative interventions while PC professionals focus on symptom management. This tension between priorities can mean that PC referrals are only made when all other options are exhausted [24, 28, 36]. Additionally, HCPs and patients might not always agree on the importance of extending life versus managing symptoms [39]. This can be addressed through more open EoL discussions and advanced care planning, which involves both PC and cardiology professionals. Finally, one review pointed out that HCPs have many competing demands in their clinical work, which may act as a barrier to engaging in advanced care planning [44].

### Mission Alignment

The reviews noted that the adoption of PC approaches aligns well with an increased use of patient-centered approaches, where care is personalized based on patients’ symptoms, needs, wishes, and preferences [30–32, 40, 41, 50]. This is due to PC’s emphasis on symptom monitoring, patient comfort, and quality of life. The increasing use of shared decision-making, patient involvement, and goal setting by healthcare organizations was also thought to align well with the goals and practices of PC [40, 44, 50]. Finally, there is an increasing use of home-based care, which aligns with healthcare organizations’ goals to move care into community settings and relieve the demand on inpatient care [30, 32, 38]. Over time, discussions of patient-centered care have remained consistent, but shared decision-making has become more commonly discussed in more recent reviews. This is likely reflective of a shift toward patient engagement and involvement in care.

### Available Resources

Several reviews discussed a lack of trained PC professionals and large workforce gaps as a barrier to meeting the demand for PC care [24, 25, 27, 30, 31, 35, 37, 38, 44, 49], particularly in rural settings [49], and in healthcare systems that rely on specialist PC professionals. The reviews also highlighted how the overburdened and stretched specialist PC workforce does not have time to deliver meaningful action planning or EoL conversations with patients [26, 27, 41, 44, 46, 47]. These issues can be addressed by making use of primary PC services in which care is provided by non-PC specialist multidisciplinary teams, including cardiologists. Using primary PC means that PC care can also be delivered earlier in the disease trajectory [44, 46, 47]. Beyond workforce gaps, several reviews discussed the lack of procedures and guidelines to support PC for patients with HF, including the lack of referral guidelines that exist in care settings, limited space in which to provide PC, and inconsistent use of mHealth and telehealth tools and technologies [24, 27, 34, 37, 38, 42–44, 46, 50]. Being able to provide a range of treatment options appropriate to the patient’s phase of HF [34] and protecting time for PC consultations were considered facilitators of care [28, 44]. Issues relating to the lack of available staff, resources, and time have been consistently referred to over the time in which these reviews were published. Despite this picture of limited availability of services, some reviews also highlight the underutilization of existing specialist PC teams in some areas [26, 52].

### Access To Knowledge and Information

The reviews discussed a lack of awareness and knowledge about PC treatment options, processes, purpose, and preferences among cardiologists, and a lack of training in HF management among PC professionals [24, 25, 27, 30, 35, 36, 38, 44, 45]. There was recognition in cardiology that specialist PC plays an important contributory role, but concerns were raised about the level of knowledge or experience that specialist PC professionals had regarding advanced HF management [47]. Additionally, there is a lack of trained PC staff and a need for PC training for the wider workforce to fill the gaps [31, 44]. Several reviews also pointed out a lack of training related to communication skills for those approaching EoL, particularly in initiating EoL conversations [40, 44, 46]. Healthcare organizations would benefit from providing education to cardiologists about PC to build understanding of the goals of care, shared decision-making, communication, experiential learning, and the intellectual and emotional demands of PC [26, 28–30, 32, 35, 36, 38, 41, 43, 44]. PC professionals would benefit from training on HF needs, including prognostication, implanted devices, and goals of care [35, 51]. Over the time in which reviews were published, the training needs for HCPs in PC and HF have remained stable; however, in more recent reviews, there has been a greater emphasis on providing non-PC specialists with PC training, indicating a shift toward the use of primary or generalist PC.

## Discussion

We have highlighted twenty-nine review articles focusing on the implementation barriers and facilitators to the implementation of PC for HF patients. These reviews have been published between 2007 and 2025 with the pace of publication increasing within that time. The review is timely because of the publication of several relevant review articles in the field between 2024 and 2025 and because of the upcoming implementation of a large EU project known as RAPHAEL which will begin in 2026. Using the CFIR, we have analyzed the barriers and facilitators to the implementation of PC in the Outer and Inner settings of twenty-nine published review articles and provided descriptions for the framework’s sub-constructs. In the Outer setting, complexity regarding the needs of patients, as well as attitudes and values towards PC, were identified as the main implementation determinants. In the Inner setting, the key implementation determinants were relational characteristics, which included communication between HCPs, communication with patients, the culture of healthcare organizations, and access to training and resources for PC.

### Improving Communication

The findings suggest that open and honest communication, shared decision-making, spiritually and culturally tailored support, compassionate care, and early engagement facilitate good PC for patients with HF and their informal caregivers. These findings align with national and international guidelines that emphasize the importance of ensuring patients and informal caregivers are adequately informed and involved in decisions surrounding EoL care [3]. However, in practice, the reviews frequently highlighted that this type of communication with patients and informal caregivers was lacking. The literature underscores the difficulty HCPs face in recognizing the patient’s palliative needs, which can lead to a lack of timely PC. Additionally, patients and caregivers have expressed the need for better communication, collaboration, and coordination of care throughout the disease pathway [53].

The reviews highlighted the value and importance of joint working in interdisciplinary teams of cardiologists and PC professionals on case management and shared projects and initiatives. Wider literature shows that interdisciplinary working between HCPs can improve care by bridging professional, social, physical, and task-related gaps, negotiating overlaps in roles and tasks, and creating spaces that allow HCPs from different disciplines to contribute in unique ways [54]. Our review also emphasizes the importance of addressing culture within healthcare organizations, particularly regarding the difference in care philosophy between cardiologists and PC professionals. Cardiologists typically focus on curative interventions, while PC professionals prioritize symptom management, comfort, and quality of life. Education on the function and values of PC can help address this cultural divide [55]. We also note that many of the issues relating to culture, collaboration, and communication with patients have been consistently discussed over the 19 years between the publication of the oldest and most recent articles in this review. That being said, we observed that more recent reviews focused more on primary PC models than on specialist PC. These discussions were often framed as a response to resource limitations in specialist care.

### Prognostic Challenges and the Complexity of Care

The complex and unpredictable trajectory of HF makes providing clear action plans for PC challenging, and patients may have misperceptions about their prognosis and need for PC. Unlike life-limiting illnesses, HF has a highly unpredictable disease course, with long periods of stability and seemingly good health, followed by steep and unpredictable declines leading up to death [56]. This complexity is compounded for patients using a cardiac device, for whom the device can introduce various challenges, including impacts on quality of life and ethical and legal concerns regarding EoL care [57]. To improve care, HCPs can focus on enhancing symptom monitoring for patients with HF to better predict and anticipate potential deterioration in their condition [58]. This can be facilitated remotely using mHealth tools for symptom tracking [59]. Another challenge to prognostication is the misperception among patients regarding the seriousness of HF, as they often do not consider their condition to warrant EoL care. In cardiology, PC is sometimes viewed as a “near-death” service that is more suited to cancer patients [60]. Improved training and interdisciplinary learning are seen as crucial steps toward changing these perceptions among both HCPs and patients.

### Workforce and Training

Across several reviews, there was acknowledgment of the lack of a trained workforce of specialist PC professionals to meet the demands of cardiac patients. Many reviews emphasized the importance of primary PC, where non-specialists are trained in PC principles and practices. This allows them to deliver PC, make better assessments, and refer patients to specialist PC services. Such an approach can facilitate earlier integration of PC into care, provide better continuity, and improve comfort and quality of life for patients, while also reducing the burden on specialist staff.

While there are many models of PC for patients with HF, a wealth of evidence supporting its efficacy, and good academic knowledge about the determinants of care, practical challenges remain, and there is a lack of evidence showing best practices for implementation. Several reviews pointed out that cardiologists do not have sufficient tools or guidelines to support EoL care or palliative referrals. In cancer care, there is a strong consensus on the value of PC, clearer referral and screening criteria, and a better understanding of implementation factors compared to HF care [61].

Finally, training needs for both PC professionals and cardiologists were highlighted as an important facilitator for implementing PC. Patient-centered and home-based care were identified as shared goals for both professions. Patient-centered care involves tailoring care to the specific symptoms, needs, concerns, and wishes of patients through shared decision-making and patient involvement [62]. The more recent reviews focused on adopting a generalist/specialist approach where all healthcare providers who treat patients with incurable illness should be able to provide generalist PC.

### Changes in the Field Over time

Many of the barriers highlighted in the reviews remained relatively stable over time. Challenges such as the complexity of the disease trajectory are difficult to address through implementation strategies as they are to some degree inherent in the illness. However, the reviews are consistent in their discussion of educational and training needs both for patients and HCPs even though this is an area where healthcare organizations can develop and deliver strategies to affect change. They focus on models of care changes throughout the reviews and the papers discussed within them. In some of the earlier reviews the focus was more on specialist PC and problems were framed around the shortage and resources of specialist PC teams. In the 2010 s and early 2020 s the focus was placed more on primary PC models and multidisciplinary working and the even more recent reviews (particularly those from 2024 to 2025) focus heavily on the delivery of home-based and community-based care. This likely reflects the shift away from hospital to community care to address the high costs and resources involved in secondary health care [63]. Finally, one of the most noticeable methodological changes over time relates to the use of Implementation frameworks to evaluate the barriers and facilitators to implementation in the reviews. Whilst the vast majority of included reviews make reference to implementation when discussing their barriers and facilitators very few actually used well recognized frameworks and theories from implementation science until 2025 in which three of the included articles [30, 33, 38] use either the Capability, Opportunity, Motivation and Behavior (COM-B) [64] model or Theoretical Domain Framework (TDF) model [65].

### Strengths and limitations

Although conducting a systematic review might have provided the authors with more targeted insights into the literature, a rapid synthesis of reviews offered a more comprehensive overview of the complexities of PC for patients with HF [66]. We used some rapid reviews methods including several simplifications made to the methodology to expedite the review process which can be seen as a limitation of our approach such as restricting the timeframe, limiting the number of databases at the full-text stage, and omitting a quality appraisal, using only one additional reviewer of articles (with input from other authors), and not conducting critical appraisal of included articles [17] (see appendix 1). We also chose to include non-systematic reviews in our final articles. Rapid review methods were used because the goal of the review was to conduct rapid and high-level evidence synthesis in a timely manner so the results can contribute to an ongoing EU research project on PC for HF patients known as RAPHAEL. These methodological adjustments to the systematic approach may impact on the robustness of our findings. This decision was informed by our goal to present an assessment of the current scholarly knowledge in the field in a timely manner with a broad scope.

### Conclusion

This review provides a broad overview of the current state of research into barriers and facilitators to the implementation of PC for patients with HF by highlighting and synthesizing insights from twenty-nine review articles using the CFIR implementation framework. The reviews highlight a wide range of barriers and facilitators which can be broadly summarized into - *Prognostic and complexity challenges*: in which the unpredictable nature of HF acts as a barrier to PC; *Communication and integrated teams*: in which multidisciplinary working, open-communication, and shared decision making can act as a facilitator to care; *Workforce and resources*: in which education and training for PC specialists, cardiologists and patients is highlighted as a facilitator whilst a lack of trained professionals, resources, and clinical time are listed as barriers. PC for HF is also changing as a field with more focus on primary or integrated PC models which make use of community-based or home-based practices as well as a greater focus on implementation science approaches for evaluating research.

## Key references

- Hatzikiriakidis K, Ayton D, Skouteris H, Patitsas L, Smith K, Dhulia A, Poon P. A rapid umbrella review of the literature surrounding the provision of patient-centered end-of-life care. Palliative medicine. 2023; 37(8):1079-99. 10.1177/02692163231183007.

This is an existing systematic umbrella literature review of palliative care services and end-of-life care which served as the basis for our search strategy. This review assesses palliative across multiple conditions and does not assess implementation barriers and facilitators. Our paper looks at the implementation barriers and facilitators to palliative care for patients with heart failure specifically.

- Damschroder LJ, Reardon CM, Widerquist MA, Lowery J. The updated Consolidated Framework for Implementation Research based on user feedback. Implementation science. 2022;17(1):75.

We made use of the Consolidated Framework for Implementation Research (CFIR) which is detailed in this paper. CFIR is primarily used to review intervention or policy implementations rather than preexisting literature. Therefore, we only used the Inner and Outer setting components of the framework to assess the literature.

- Nilsen P (2020) Making sense of implementation theories, models, and frameworks. In: Implementation Science 3.0. Springer, pp 53–79.

We selected CFIR to review the literature based on this methodological paper which defines, compares and describes applications of a wide range of implementation frameworks. The author defines CFIR as a ‘determinant framework’ meaning that it is well suited to identifying barriers and facilitators to implementation. This is aligned with the goals of this paper.

## Data Availability

No datasets were generated or analyzed during the current study.

## Appendix 1

**Table 4:** Methodological simplifications to the review process

**Common streamlining methods** (identified by Tricco et al. 2015)	**Streaming methods used in this study (Yes**,** they were used – No**,** they were not used)**
Not publish a protocol	We did not publish a protocol for this review.
Review question	Our review question is clearly presented.
Limiting the literature search to published literature	We limited the search to published literature. We did not assess grey literature.
Limiting the literature search to one database	We conducted the literature search in four comprehensive and relevant databases including: Scopus, EMBASE, CINAHL, and Cochrane
Limiting inclusion criteria by date	We did not limit the search by date.
Limiting the literature search based on language	We limited the literature search to English language papers.
Having one person screen and another verify, or screen excluded studies.	We had one person screen the articles whilst one other researcher verified the included studies, the studies labelled as “maybe” and a selection of excluded articles.
Having one-person extract data and another verify	One person extracted the data whilst two other researchers verified the abstraction.
Not conducting risk of bias/quality appraisal	We did not conduct a risk of bias/quality appraisal.
Presenting results as a narrative summary	We conducted a literature synthesis based on the CFIR framework for implementation research.
Not contacted authors for reviews that could not be found	We attempted to contact authors of reviews that we could not find online via email.

## Appendix 2

**Table 5:** Search terms for the review

Search concepts	Search terms
Heart Failure	‘Heart Failure’ OR ‘Cardiac decompensation’ OR ‘heart decompensation’ OR ‘cardiac dysfunction’ OR ‘heart dysfunction’ OR ‘systolic dysfunction’ OR ‘diastolic dysfunction’ OR ‘ventricular dysfunction’ OR ‘HF’
Palliative care	‘Palliative care’ OR ‘palliative therapy’ OR ‘Palliative medicine’ OR ‘Palliative’ OR ‘palliation’ OR ‘terminal care’ OR ‘hospice care’ OR hospice OR ‘advance care planning’ OR ((‘terminal’ OR ‘hospice’ OR ‘supportive’ OR ‘end-of-life’ OR ‘dying’) AND care)
Implementation	‘routin*’ OR ‘normali*’ OR ‘integrat*’ OR ‘facilitat*’ OR ‘barrier*’ OR ‘Implement*’ OR ‘adopt*’
Reviews	‘review*’ OR ‘literature’ OR ‘evidence synthesis’

## Appendix 3

**Table 6:** Inclusion and Exclusion criteria

Inclusion Criteria	Exclusion Criteria
(i) were published in the English language	(i) constituted grey literature
(ii) constituted outputs of secondary evidence synthesis of academic literature sources	
(iii) were published in a peer-reviewed journal	
(iv) reported on barriers and facilitators to implementation associated with PC	
(v) focused on advanced HF patients	
